# The cancer-associated fibroblasts interact with malignant T cells in mycosis fungoides and promote the disease progression

**DOI:** 10.3389/fimmu.2024.1474564

**Published:** 2025-02-03

**Authors:** Yige Zhao, Yong Li, Panpan Wang, Mengyan Zhu, Jiaqi Wang, Bo Xie, Chenyu Tang, Yangyang Ma, Shiwen Wang, Sha Jin, Jinhui Xu, Zhao Li, Xiaoyan Zhang, Liuyu Li, Xiuzu Song, Ping Wang

**Affiliations:** ^1^ Department of Dermatology, Hangzhou Third People's Hospital, Hangzhou Third Hospital Affiliated to Zhejiang Chinese Medical University, Hangzhou, China; ^2^ Research Center, Shanghai Yeslab Biotechnology, Shanghai, China; ^3^ Department of Dermatology, Shaoxing People's Hospital, Shaoxing, China; ^4^ Department of Dermatology, Hangzhou Third People's Hospital, Zhejiang Chinese Medical University, Hangzhou, China

**Keywords:** mycosis fungoides (MF), cutaneous T cell lymphoma (CTCL), tissue resident memory T cell (TRM), cancer-associated fibroblast (CAF), tumor microenvironment, SOX4

## Abstract

**Background:**

Cutaneous T-cell lymphoma (CTCL) is a heterogeneous group of T-cell lymphomas characterized with the presence of clonal malignant T cells. Mycosis fungoides (MF) is the most common type of CTCL. However, the pathogenesis of MF and the role of the tumor microenvironment (TME) remain unclear.

**Methods:**

We performed single-cell RNA sequencing on tumor and adjacent normal tissues and peripheral blood mononuclear cell (PBMC) from patients with advanced MF and healthy control (HC). We compared skin lesions in different stages within the same patient to overcome inter-individual variability.

**Results:**

The malignant clones displayed dual phenotypes characterized with tissue-resident memory T cells (TRMs) and central memory T cells (TCMs). We supposed that the tumor cells transformed from TRM-dominant phenotype to TCM-dominant phenotype during MF progressed from early-stage to advanced-stage. The cancer-associated fibroblasts (CAFs) showed active role in TME. The occurrence of inflammatory CAFs (iCAFs) may represent the advanced-stage MF. There may be mutual positive feedback of the crosstalk between tumor cells and CAFs during the MF development. Tumor cells promote CAF generation, and the CAFs, in turn, improve the invasiveness and metastasis of the malignant T cells through the IL-6/JAK2/STAT3/SOX4 or IL-6/HIF-1α/SOX4 pathway. SOX4 may be a critical regulatory gene of this positive feedback loop. Target SOX4 may disrupt the interactions between tumor cells and CAFs.

**Conclusion:**

Our study revealed the origin and evolution trajectory of MF and uncovered the intercellular interactions between malignant T cells and CAFs, providing new insights into the novel treatment targets of MF.

## Introduction

Primary cutaneous T-cell lymphomas (CTCLs) are groups of peripheral non-Hodgkin’s lymphomas. Mycosis fungoides (MF) is the most common type of CTCL, accounting for approximately 53% of all cutaneous lymphomas (1). MF is characterized by a unique clinical course and a relatively indolent biologic behavior in the early stage. It progresses slowly over years or even decades, presenting in the form of patches, plaques, and, then, tumors. The disease could invade peripheral blood, lymph nodes, or viscera (1) eventually. The general 5-year survival rate of MF is 70%–80%; however, 5%–55% of patients undergo large-cell transformation during the disease process. The prognosis of this part of patients is poor, with an average survival period of 2 to 36 months (2). It supposed that neoplastic T cells in MF derived from mature, monoclonal, and skin-resident memory T cells (TRMs) (3). However, recent studies suggest that malignant T cells are derived from immature circulating precursor cells (4–6). The origins and development trajectory of MF remain controversial.

More studies have been focused on elucidating the roles of tumor microenvironment (TME) in the development and progression of CTCL. Rindler et al. (7) explored the TME of patients with advanced MF. Moreover, a molecular subtyping scheme for malignant T cells was reported in previous study (8). They also deciphered the complex crosstalk among immune cells for each subtype within the TME. Du et al. reported a landscape of immunosuppressive TME mediated by interactions between malignant T cells and myeloid cells (9). However, the role of other TME components in MF, such as fibroblasts, endothelial cells, and B cells, has not been fully understood.

In order to study the role of cancer-associated fibroblasts (CAFs) in TME during MF development, we performed single-cell transcriptomic analysis on eight samples from two patients with advanced MF and a matched healthy control (HC). Copy number variations (CNVs) and pseudotime analysis were applied to define malignant subclones and track the progression trajectory of malignant T cells in MF.

Our analysis revealed the origin and phenotypic changes of malignant T cells during the evolution of MF. Four CAF subclusters in MF and adjacent tissues revealed their changes of gene expression at different stages of the disease. The complex interaction between malignant T cells and fibroblasts illustrated the role of tumor cells in TME remodeling and the influence of CAFs on tumor progression.

## Methods

### Patient recruitment and sample processing

Patients were recruited from the Skin Lymphoma Clinic of Hangzhou Third People’s Hospital, China (**Table 1**). All patients included did not receive any topical or systemic therapies in the past 6 months prior to biospecimen collection. All diagnoses were verified by at least two dermatopathologists according to World Health Organization-European Organization for Research and Treatment of Cancer (WHO-EORTC) classification criteria (10). We took biopsies from both flat and palpable lesion at the same time from two patients with MF. Additionally, the corresponding adjacent non-lesional skin and PBMCs were collected in MF1. Each freshly dissociated sample was transported to laboratory immediately. These MF skin tissues together with the HC sample were performed single-cell RNA sequencing (scRNA-seq). Data were analyzed using the R package Seurat (11). All samples were obtained after informed consent and approval from the Medical Ethics Committee of Hangzhou Third hospital.

**Table 1:** The basic information of the patients at time of sampling.

Patient	Subject ID	Stage	Age	Sex	Disease stage
MF1	MF1_b1	Plaque	69	Male	T4N1M0B0(IIIA)
	MF1_b2	Adjacent non-lesional skin of MF1_b1			
	MF1_c1	Tumor			
	MF1_c2	Adjacent non-lesional skin of MF1_c1			
	PBMC1	–			
MF2	MF2_a1	Patch	75	Male	T4N1M0B0(IIIA)
	MF2_c1	Tumor			
HC	HC	Healthy control skin	62	Male	–

MF, mycosis fungoides; PBMC, peripheral blood mononuclear cell; HC healthy control.

### Preparation of single-cell suspensions

Each sample was subsequently minced on ice to less than 1-mm cubic pieces, followed by enzymatic digestion. Samples were then centrifuged at 300 relative centrifugal force (rcf) for 30 s at room temperature and removed the supernatant without disturbing the cell pellet. Next, 1× Phosphate Buffered Saline (PBS) (calcium and magnesium free) containing 0.04% weight/volume Bovine Serum Albumin (BSA) (400 µg/mL) was added and then centrifugation at 300 rcf for 5 min. The cell pellets were resuspended in 1 mL of red blood cell lysis buffer and incubated for 10 min at 4°C. After red blood cell lysis, samples were resuspended in 1 mL of PBS containing 0.04% BSA. Next, samples were filtered over Scienceware Flowmi 40-µm cell strainers (VWR). After tumor dissociation, cell concentration and cell viability were determined by hemocytometer and Trypan Blue staining.

### Single-cell RNA-seq data preprocessing

The Cell Ranger software pipeline (version 5.0.0) provided by 10× Genomics was used to demultiplex cellular barcodes, map reads to the genome and transcriptome using the STAR aligner, and down-sample reads as required to generate normalized aggregate data across samples, producing a matrix of gene counts versus cells. We processed the unique molecular identifier (UMI) count matrix using the R package Seurat (11) (version 3.1.1). To remove low quality cells and likely multiple captures, which is a major concern in microdroplet-based experiments, we applied a criterion to filter out cells with gene numbers less than 200, UMI less than 1,000, and log10GenesPerUMI less than 0.7. We further discarded low-quality cells where >20% of the counts belonged to mitochondrial genes and >5% of the counts belonged to hemoglobin genes. Additionally, we applied DoubletFinder package (12) (version 2.0.2) to identify potential doublet. After applying these QC criteria, 42,480 single cells were included in downstream analyses. Library size normalization was performed with NormalizeData function in Seurat (11) to obtain the normalized count. Specifically, the global-scaling normalization method “LogNormalize” normalized the gene expression measurements for each cell by the total expression, multiplied by a scaling factor (10,000 by default), and the results were log-transformed.

Top variable genes across single cells were identified using the method described in Macosko et al. (13). The most variable genes were selected using FindVariableGenes function (mean.function = FastExpMean, dispersion.function = FastLogVMR) in Seurat (11). To remove the batch effects in single-cell RNA-sequencing data, the mutual nearest neighbors (MNNs) presented by Haghverdi et al. were performed with the R package batchelor (14). Graph-based clustering was performed to cluster cells according to their gene expression profile using the FindClusters function in Seurat (11). Cells were visualized using a two-dimensional Uniform Manifold Approximation and Projection (UMAP) algorithm with the RunUMAP function in Seurat (11). We used the FindAllMarkers function (test.use = presto) in Seurat (11) to identify marker genes of each cluster. For a given cluster, FindAllMarkers identified positive markers compared with all other cells. Then, we used the R package SingleR (15) (version 1.4.1), a novel computational method for unbiased cell-type recognition of scRNA-seq, with the reference transcriptomic datasets “Human Primary Cell Atlas” (16) to infer the cell of origin of each of the single cells independently and identify cell types.

### Copy number variation analysis

To estimate the initial CNVs for each region, we utilized the inferCNV (17) R package. The CNV of total cell types was calculated on the basis of the expression level derived from single-cell sequencing data for each cell, with a cutoff of 0.1. Genes were sorted according to their chromosomal location, and a moving average of gene expression was computed with a window size of 101 genes. The expression values were then centered to zero by subtracting the mean. The T cells were considered as malignant cells, whereas all other cells were considered normal cells. De-noising techniques were applied to generate the final CNV profiles.

### Differentially expressed gene analysis

Differentially expressed genes (DEGs) were analyzed using the FindMarkers function (test.use = presto) in Seurat (11). P-value < 0.05 and |log2foldchange| > 0.58 were set as the threshold for significantly differential expression. Gene Ontology (GO) enrichment and Kyoto Encyclopedia of Genes and Genomes (KEGG) pathway enrichment analysis of DEGs were respectively performed using R based on the hypergeometric distribution.

### Pseudotime analysis

To determine the developmental pseudotime, we utilized the Monocle2 package (18). The raw count was first converted from Seurat object into CellDataSet object with the importCDS function in Monocle. The differentialGeneTest function of the Monocle2 package was used to select ordering genes (qval < 0.01), which were likely to be informative in the ordering of cells along the pseudotime trajectory. We performed dimensional reduction clustering analysis using the reduceDimension function, followed by trajectory inference using the orderCells function with default parameters. Gene expression was plotted with the plot_genes_in_pseudotime function to track changes over pseudotime.

### Gene set variation analysis

To conduct the gene set variation analysis (GSVA), we utilized the GSEABase package (version 1.44.0) to load the gene set file. The gene set file was downloaded and processed from the KEGG database (https://www.kegg.jp/). For assigning pathway activity estimates to individual cells, we employed GSVA (19) with standard settings, implemented in the GSVA package (version 1.30.0). The differences in pathway activities per cell were calculated using the LIMMA package (version 3.38.3).

### SCENIC analysis

The SCENIC analysis was conducted using the motifs database for RcisTarget and GRNboost (SCENIC (20) version 1.1.2.2, which corresponds to RcisTarget 1.2.1 and AUCell 1.4.1) with the default parameters. In detail, we identified transcription factor (TF)–binding motifs over-represented on a gene list with RcisTarget package. The AUCell package was used to score the activity of each group of regulons in each cell. The connection specificity index for all regulons was calculated with the scFunctions (https://github.com/FloWuenne/scFunctions/) package.

### Cell–cell communication analysis

The CellPhoneDB (21) (v2.0) was performed to detect biologically relevant ligand–receptor (LR) interactions from single-cell transcriptomics (scRNA-seq) data. We defined a ligand or a receptor as “expressed” in a particular cell type if 10% of the cells of that type had non-zero read counts for the ligand/receptor encoding gene. We evaluated the statistical significance of these interactions by shuffling the cluster labels of all cells and repeating the above steps, producing a null distribution for each LR pair in pairwise comparisons between two cell types. After running 1,000 permutations, P-values were calculated with the normal distribution curve generated from the permuted LR pair interaction scores. To establish networks of cell–cell communication, we connected any two cell types where the ligand was expressed in the former cell type and the receptor in the latter. R packages Igraph and Circlize were used to visualize the cell–cell communication networks.

### Immunohistochemistry and immunofluorescence

Paraffin-embedded tissue microarray was dewaxed and rehydrated to retrieve the antigens. The sections were incubated with primary antibodies at 4°C overnight. After washing with PBS, the horse radish peroxidase–conjugated secondary antibody was added and further incubated for 2 h. The diaminobenzidine (Beyotime) kit was used to visualize antibody binding. After washing, nuclei were stained with hematoxylin. Primary antibodies used in immunohistochemistry (IHC) and immunofluorescence (IF) are listed as follows: cluster of differentiation 4 (CD4) (1:100, Abcam, ab133616, clone EPR6855), CD8 (1:1,000, Proteintech, 66868-1-lg, clone 1G2B10), SRY-related high-mobility-group box 4 (SOX4) (1:200, CUSABIO, CSBPA022431LA01HU), Nuclear Receptor Subfamily 4 Group A Member 1 (NR4A1) (1:200, Proteintech, 25851-1-AP), CD103 (1:100, Abcam, ab224202, EPR22590-27), Fibroblast Activation Protein Alpha (FAP) (1:250, Abcam, ab207178, EPR20021), Twist Family BHLH Transcription Factor 1 (TWIST1) (1:200, Proteintech, 25465-1-AP), Matrix Metallopeptidase 2 (MMP2) (1:200, Proteintech, 10373-2-AP), and Interleukin 6 (IL-6) (1:200, Proteintech, 21865-1-AP).

### Survival analysis

The Gene Expression Omnibus (GEO) gene expression and survival data were downloaded from the National Center for Biotechnology Information (NCBI) ‘s GEO (22) and are accessible through GEO series accession number GSE168508 (https://www.ncbi.nlm.nih.gov/geo/query/acc.cgi?acc=GSE168508). The samples were grouped into high and low groups based on the optimal cut point determined by R function surv_cutpoint. Kaplan–Meier survival curves were plotted by survminer package.

**Illustration tool:** **Figure 7** was created with BioRender.com (https://biorender.com).

## Results

### Single-cell RNA sequencing revealed the cell profile in MF

**Figure 1:**
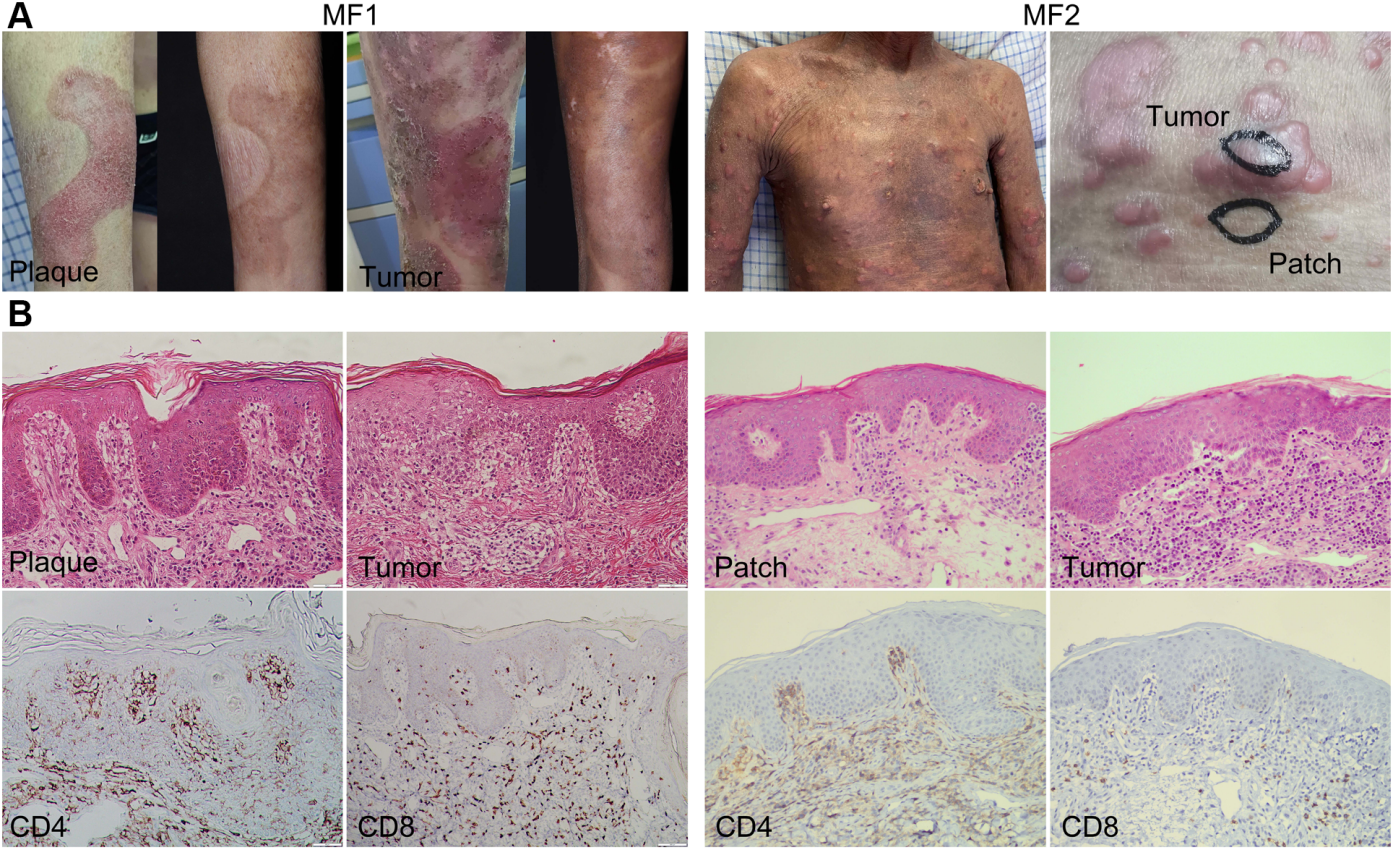
Clinical and histopathological pictures of two patients with MF. **(A)** Clinical pictures of MF lesions biopsied for single-cell RNA sequencing. The pictures of MF1 displaying the lesion in the same location before and after treatment. **(B)** Representative histopathological pictures of MF lesions from two patients (biopsies for initial MF diagnosis).

**Figure 2:**
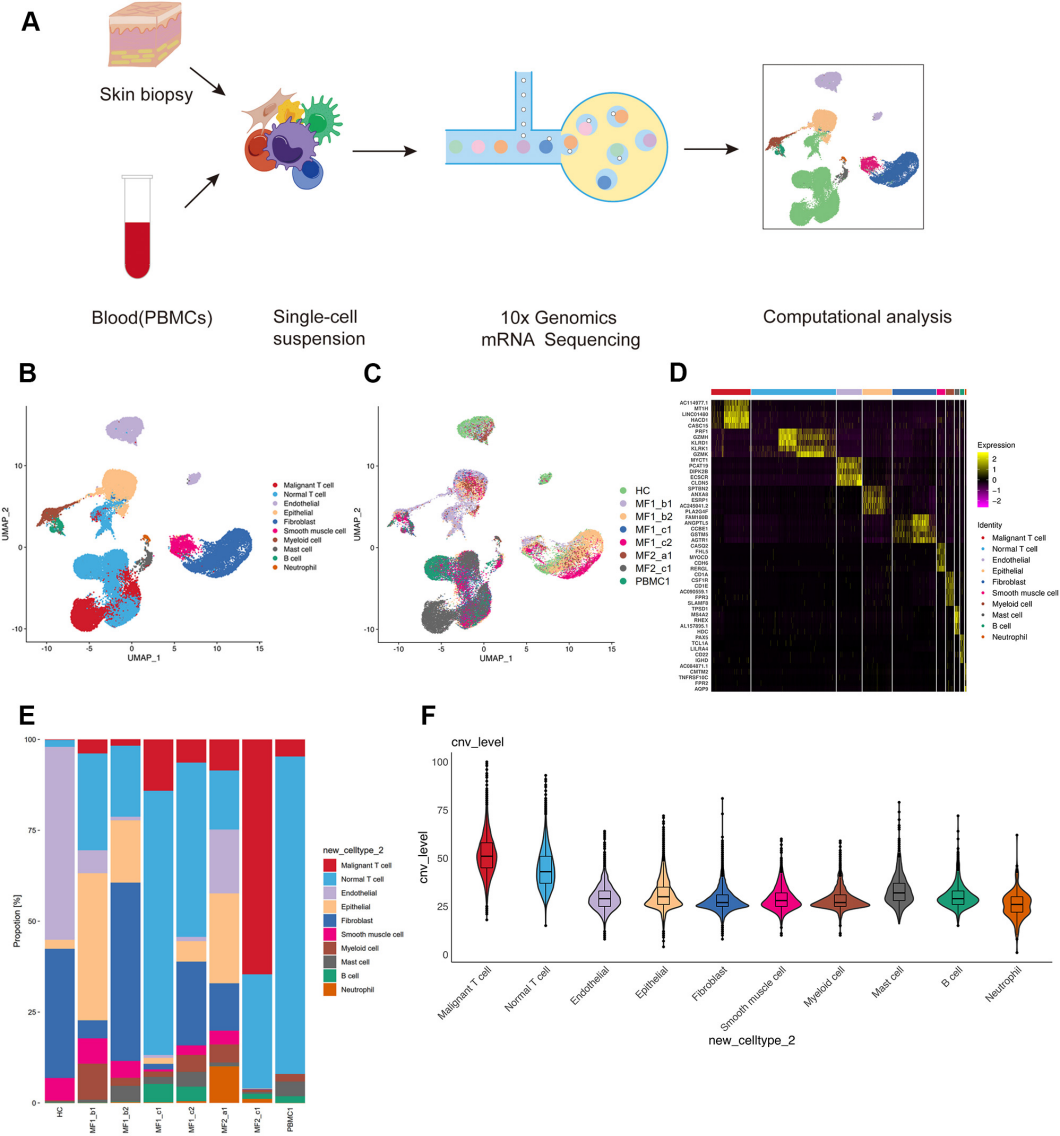
Single-cell transcriptional profiling of patients with MF and HC. **(A)** Workflow of sample collection, single-cell dissociation, cell sorting, and computational analysis for scRNA-seq data. **(B)** UMAP visualization of 42,480 single cells from two patients with MF and one HC. **(C)** UMAP visualization of 10 cell types sourced from each sample. **(D)** The heatmap displays the top five significantly differentially expressed (SDE) genes in each cell type. **(E)** Stacked histogram showing the percentage of cells from various cell types in each sample. **(F)** Violin plots show CNV levels among 10 cell types.

We collected and analyzed the scRNA-seq data of eight samples from one HC and two patients with advanced-stage MF without treatment, including one MF (MF1) and one patient with large cell transformation (MF2) (**Figure 1A**; **Table 1**). The two patients were confirmed by histopathology examination (**Figure 1B**). MF1 was followed up, and the skin lesions resolved partially after 6 months of treatment (**Figure 1A**). We collected four skin lesions in different stages and two adjacent tissues from patients with MF, together with a peripheral blood mononuclear cell (PBMC) sample from MF1 before treatment. Matched non-cancer skin sample was collected form a HC. In total, 42,480 individual cells from eight samples were passed quality control criteria (**Supplementary Table 1**), which were unsupervisedly clustered into 17 major clusters after scRNA-seq (**Figure 2A**). These clusters were visualized by UMAP, with the MNNs employed to remove the batch effects (**Supplementary Figure 1A**).

According to the specifically expressed genes in each cluster, 10 cell types were annotated (**Figure 2B**; **Supplementary Figure 1B**), including B cells (653 cells), endothelial cells (4,101 cells), epithelial cells (4,761 cells), fibroblasts (7,087 cells), mast cells (786 cells), myeloid cells (1,305 cells), neutrophils (262 cells), smooth muscle cells (1,378cells), normal T cells (13,729 cells), and malignant T cells (6,375 cells). To further confirm the annotation of clustered cells, the specifically expressed genes of each cell types were identified (**Figure 2D**). We performed large-scale CNVs analysis to define the malignant cells. Malignant T cells showed dramatically higher CNV level compared with other cell types (**Figure 2F**). Although most of the 10 cell types were found in all skin samples, the proportion of each cell type is largely varied among each sample (**Figures 2C, E**). This heterogeneity especially reflected in the differential enrichment of T cells between patients with MF and HC (**Figure 2E**).

### Transcriptomic heterogeneity of malignant T cells

**Figure 3:**
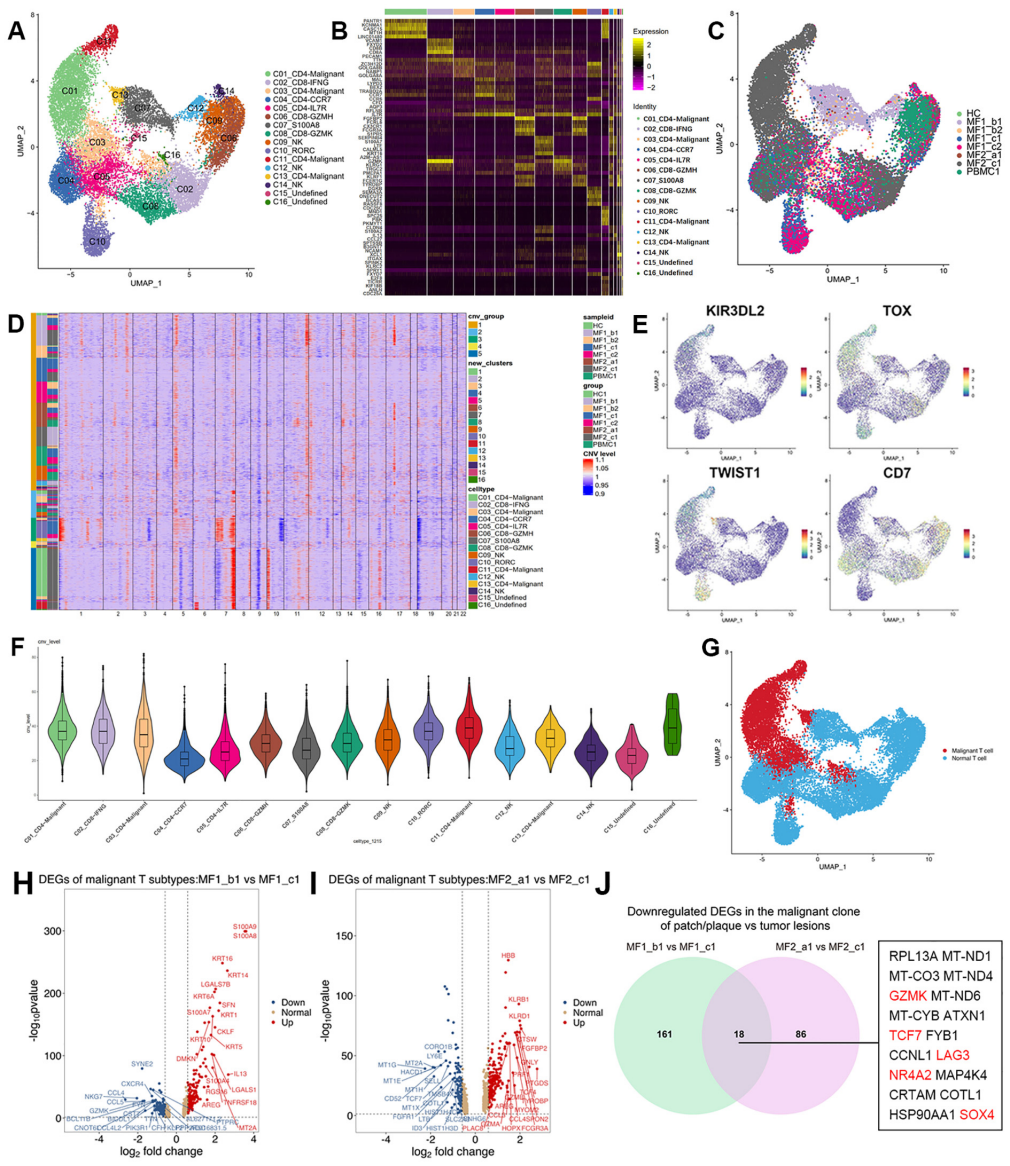
Transcriptional profiles of T-cell subpopulations in patients with MF and HC. **(A)** UMAP visualization of 16 T-cell subpopulations. **(B)** Heatmap for gene expression levels of top five SDE genes of T-cell subtypes. **(C)** UMAP visualization of the T cells sourced from each sample. **(D)** The heatmap displays large-scale CNVs of T cells. The red color represents high CNV level and blue represents low CNV level. **(E)** Expression levels of specific markers for T-cell subtypes are plotted onto the UMAP plots. **(F)** Violin plots show CNV levels among 16 T-cell types. **(G)** Malignant and normal T cells in the UMAP plot are marked by different colors, malignant T cells in red and normal T cells in blue. **(H)** Volcano plots of DEGs in malignant T cells comparing MF1_b1 (plaque) and MF1_c1 (tumor) (log2 fold change > 1.5, p < 0.05). **(I)** Volcano plots of DEGs in malignant T cells comparing MF2_a1 (patch) and MF2_c1 (tumor) (log2 fold change > 1.5, p < 0.05). **(J)** Venn diagram of significantly downregulated genes comparing gene expression in the malignant clone of patch/plaque vs. tumor lesion.

T cells from each donor were re-clustered into16 distinct cell clusters followed by visualization using UMAP (**Supplementary Figure 2A**). Furthermore, to identified the malignancy of the subpopulations, large-scale chromosomal CNVs inferred from transcriptome sequencing were examined (**Figures 3D, F**). Clusters C01, C03, C11, and C13 showed high level of CNVs, which was also displayed in the UMAP plot (**Supplementary Figure 4A**). We defined those clusters as malignant T cells (C01_CD4-Malignant, C03_CD4-Malignant, C11_CD4-Malignant, and C13_CD4-Malignant). For the rest clusters, the expressions of genes associated with typical T-cell functions were analyzed for annotation, such as C04_CD4-CCR7, C05_CD4-IL7R, and C08_CD8-GZMK (**Figures 3A, B**).

The reactive T cells from each sample showed a high degree of overlap, whereas the malignant T cells grouped into clear patient-specific clusters, demonstrating the cellular heterogeneity of malignant T cells (**Figure 3C**). The percentage of malignant T cells in the overall T-cell population varied significantly among samples, even among those from the same patient (**Supplementary Figure 2B**), adding to the complexity of inter-tumor heterogeneity among MF lesions. The disease heterogeneity in the molecular level has been indicated in gene expression among T-cell subtypes. Compared with the reactive T cells, the expression of tumor-associated genes upregulated in malignant T-cell subsets, including Killer cell immunoglobulin-like receptor 3DL2 (KIR3DL2), Thymocyte Selection Associated High Mobility Group Box (TOX), Gametocyte Specific Factor 1 (GTSF1), CD40 Ligand (CD40LG) (**Figure 3E**; **Supplementary Figure 3**). The distribution pattern of malignant and normal T cells displayed on the UMAP plot (**Figure 3G**).

### Change of cell phenotypic revealed the origin of malignant cells

**Figure 4:**
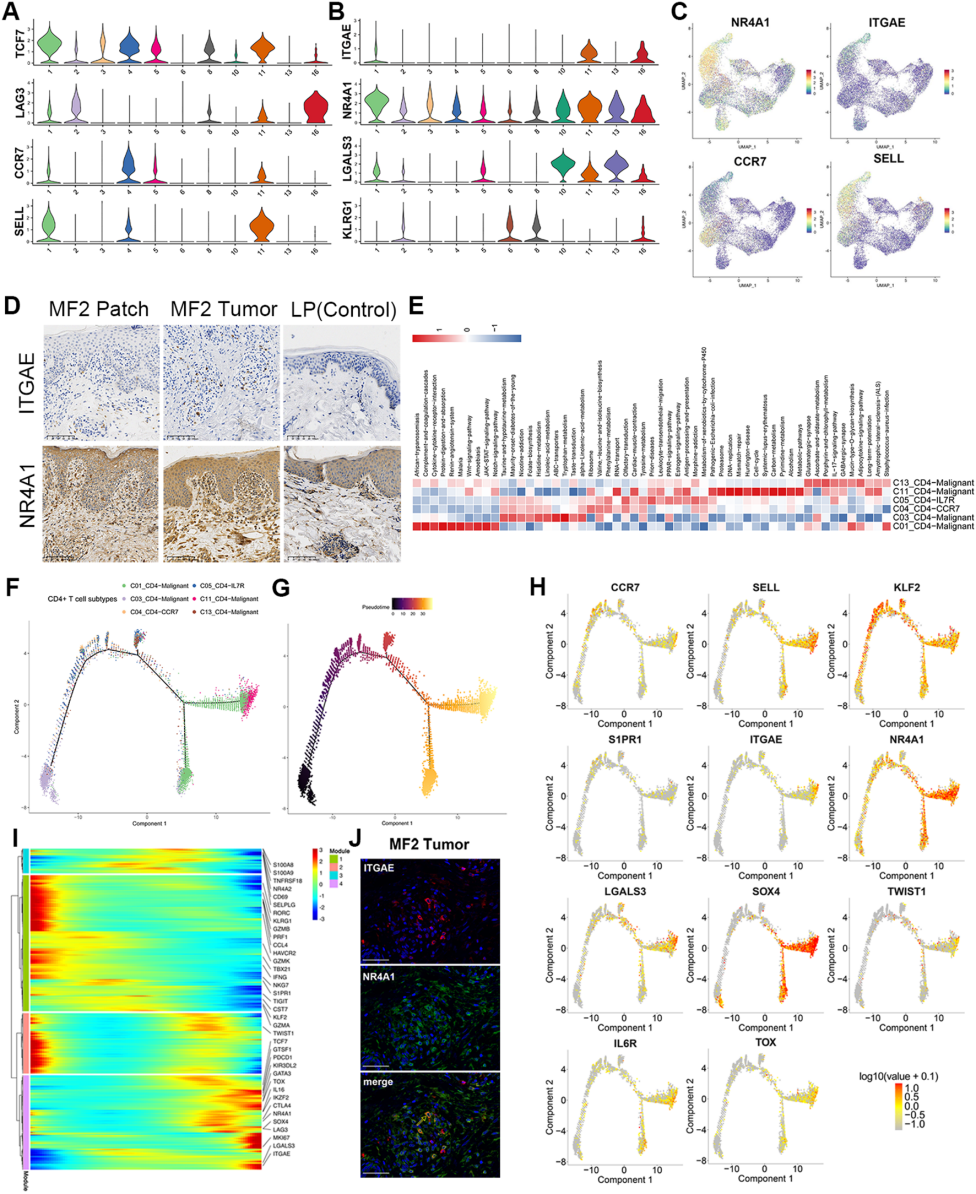
Gene regulatory networks in MF. Pseudotime trajectory analysis of CD4+ and CD8+ T cells. **(A)** Violin plots show the expression of exhausted T and TCM cell markers in T-cell subtypes (C01–C06, C08, C10, C11, C13, and C16). **(B)** Violin plots show the expression of TRM cell markers in T-cell subtypes (C01–C06, C08, C10, C11, C13, and C16). **(C)** Expression levels of specific markers for T-cell subtypes are plotted onto the UMAP plots. **(D)** Immunohistochemical stain of CD103 (ITGAE) and NR4A1 in skin biopsies of patch stage MF lesion, tumor stage MF lesion, and LP lesion. Original magnification was ×20. Scale bar, 100 μm. **(E)** GSVA analysis indicates enriched pathways of each subset of CD4+ T cell. **(F)** Density plot showing the distribution of each CD4+ T-cell subpopulation along the pseudotime inferred by analysis with Monocle2. **(G)** Reactive T cells were selected as the start cells, color key from dark to bright indicates cancer progression from the early to the late stage. **(H)** Trajectory plots with the expression of respective malignant T-cell differentiation-associated genes: highest expression in red and lowest expression in gray. **(I)** Heatmap showing the dynamic changes in gene expression along the pseudotime of CD4+ T cells. Color key from blue to red indicates relative expression levels from low to high. **(J)** Immunofluorescence staining of CD103 (ITGAE) (red), NR4A1 (green) on paraffin-embedded tissue samples of tumor stage lesion from MF2 patient. 4',6 - diamidino - 2 - phenylindole) (DAPI) (blue) was used to visualize cell nuclei. Scale bar, 50 μm.

In this study, we compared patches or plaque with tumors within the same patient to overcome inter-individual variability. We profiled the transcriptional expression patterns of all T cells and identified the DEGs in the malignant T cells in different stages of two patients (**Figures 3H, I**). Eighteen DEGs consistently upregulated in two patients with disease progression, including Transcription Factor 7 (TCF7), Lymphocyte Activating 3 (LAG3), Granzyme K (GZMK), Nuclear Receptor Subfamily 4 Group A Member 2 (NR4A2), and SOX4 (**Figure 3J**). TCF7 and LAG3, which upregulated in C01_CD4-Malignant and C11_CD4-Malignant subclusters, are the markers of precursor exhausted T cells (23) (**Figure 4A**).

NR4A2 (Nurr1) is associated with tissue retention of TRMs. Similar to NR4A2, NR4A1 (Nur77) and AHR are the genes involved in the development and function of TRM cells (24–26). NR4A1 showed high expression level in skin-derived malignant T cells (**Figures 4B, C**). In addition, Galectin 3 (LGALS3) is a new marker for human skin TRMs, which is also significantly upregulated in C01, C10, C11, and C13 subclusters (**Figure 4B**). Moreover, these clusters do not express KLRG1 (**Figure 4B**). The TRM cells originate from KLRG1-precursor cells, which begin to mature when entering into the tissues, and migrate into the epidermis via chemokine-dependent mechanisms. These precursor cells eventually acquire constitutive surface expression of CD69 and CD103 Integrin Subunit Alpha E (ITGAE), which are recognized as key markers of skin TRM cells (27).

HC and IF staining were performed to validate the expression of TRM markers, including CD103 and NR4A1 (**Figures 4D, J**). The results showed that both CD103 and NR4A1 were increased in the dermis of tumor and patch lesion in advantage-stage MF compared to lichen planus patients (**Figure 4D**). However, the circulating neoplastic cells from the PBMCs of MF1 showed lower levels of skin homing molecules genes (CCR4 and CCR10) and tissue-resident associated genes (NR4A1 and CD103) (**Supplementary Figure 2D**), compared with the skin-derived malignant T cells. Based on the above results, the gene expression pattern of skin-derived malignant T cells was similar to TRM. Furthermore, the CD4+ malignant T cells (mainly in MF2) showed high expression of TCM markers (CCR7 and SELL) (**Figures 4A, C**). GSVA analyses demonstrated that Wnt, JAK−STAT, Notch and IL−17−signaling−pathway upregulated in malignant CD4+ T cells (**Figure 4E**).

We performed trajectory analysis on CD4+ T cells to trace the origin of malignant cells (**Figures 4F–I**). The pseudotime trajectory started with the subclones containing the fewest numbers of CNVs and ended with subclones displays more extensive CNVs (**Figure 4G**; **Supplementary Figure 4B**). Consistent with the CNV results, the expression of tumor-associated genes, including KIR3DL2, TOX, TWIST1, and GTSF1, upregulated along the CD4+ T-cell trajectory (**Figure 4H**; **Supplementary Figure 4C**). By analyzing the variation of T-cell differentiation–associated genes along the pseudotime trajectory (**Figures 4H, I**), we found the TF KLF2 and its downstream target gene S1PR1 were reduced from benign to malignant CD4+ T cells (**Figures 4H, I**), which is a critical step in tissue residence and differentiation of TRM. Besides, the TCM marker of CCR7 and SELL upregulated along the CD4+ T-cell trajectory (**Figure 4H**). This is consistent with above results that the CD4+ malignant T cells display dual TRM and TCM cell phenotypes.

### Transcriptional expression patterns of CAFs in MF

**Figure 5:**
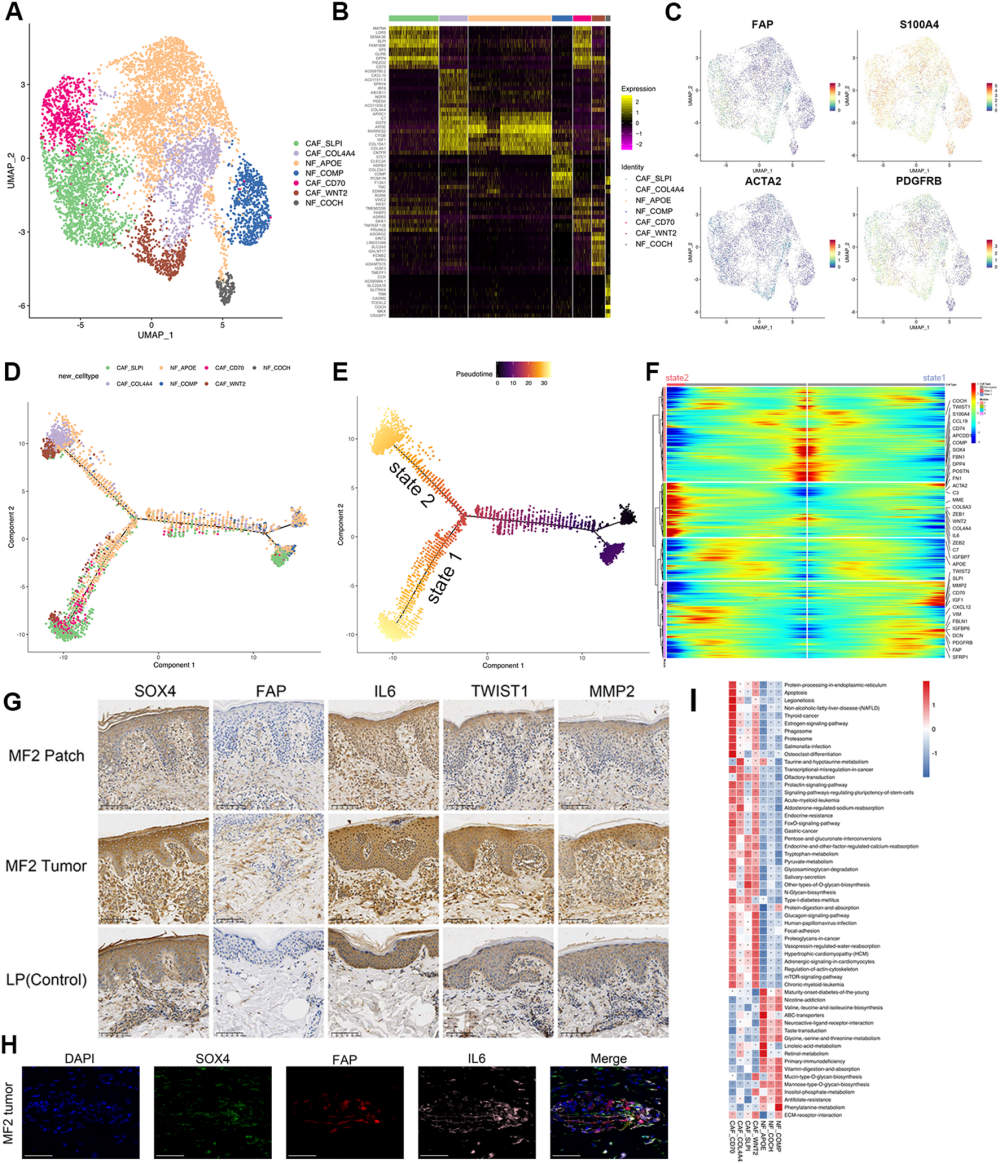
Transcriptional profiles of fibroblast subpopulations in patients with MF and HC. **(A)** UMAP visualization of seven fibroblast subpopulations. **(B)** Heatmap for gene expression levels of top 10 SDE genes of fibroblast subtypes. **(C)** UMAP plots color-coded for the expression of marker genes for CAF subtypes. **(D)** Density plot showing the distribution of each fibroblast subpopulation along the pseudotime inferred by analysis with Monocle2. **(E)** NFs were selected as the start cells, color key from dark to bright indicates cancer progression from the early to the late stage. **(F)** Heatmap showing the dynamic changes in gene expression along the pseudotime of fibroblasts. Color key from blue to red indicates relative expression levels from low to high. **(G)** Immunohistochemical stain of SOX4, FAP, IL-6, TWIST1, and MMP2 in skin biopsies of patch stage MF lesion, tumor stage MF lesion, and LP lesion. Original magnification was ×20. Scale bar, 100 μm. **(H)** Representative IF staining of FAP (red), SOX4 (green), and IL-6 (magenta) on paraffin-embedded tissue samples of tumor stage lesion from MF2 patient. DAPI (blue) was used to visualize cell nuclei. Scale bar, 50 μm. **(I)** GSVA analysis indicates enriched pathways of each subset of CD4+ T cell.

CAFs play an essential role in carcinogenesis, and it suggested that the CAFs in activated phenotypes can promote MF progression in the early stage (28). To investigate the biological characteristics of CAFs in MF, the fibroblasts isolated from each skin samples were re-clustered into nine subclusters (**Supplementary Figure 5A**). We employed the ACTA2 (alpha-smooth muscle actin 2) , FAP, S100A4, and platelet-derived growth factor receptor-β (PDGFRβ) as a set of markers to identify CAFs (**Figure 5C**) (29). According to these CAF-related makers (**Figure 5C**; **Supplementary Figure 5D**), the nine fibroblast subclusters were defined as four CAF subtypes (CAF-SLPI, CAF-CD70, CAF-COL4A4, and CAF-WNT2) and three normal fibroblast (NF) subtypes (NF-APOE, NF-COCH, and NF-COMP) (**Figures 5A, B**).

Typical fibroblast markers such as FAP, S100A4, and PDGFRβ were highly expressed in four CAF subclusters (**Figure 5C**). IHC staining of CAF marker FAP showed that the FAP-positive cells from tumor lesion were significantly increased and had a stronger staining compared to the dermis of patch lesion in the same patients (**Figure 5G**). CAFs can be recognized in all patients with MF but were particularly enriched in para-cancerous tissues (**Supplementary Figure 5C**). CAF-CD70 and CAF-SLPI were predominantly enriched in para-cancerous tissues of plaque stage (MF1_b2), whereas CAF-WNT2 and CAF-COL4A4 were mainly enriched in para-cancerous tissues of tumor stage (MF1_c2) (**Supplementary Figure 5C**). However, the distribution of NF subtypes overlapped in MF and HC samples (**Supplementary Figures 5B, C**). This heterogeneity was also observed in the pseudotime analysis of fibroblasts. The differentiation trajectories started with NFs, whereas the CAFs distributed over the two branches (state 1 and state 2) of the trajectory (**Figures 5D, E**). The CAFs on two states upregulated the extracellular matrix (ECM) signatures, including collagen molecules (COL6A3), periostin (POSTN), Fibronectin 1 (FN1), Decorin (DCN), Insulin Like Growth Factor Binding Protein 6 (IGFBP6), Secretory Leukocyte Peptidase Inhibitor (SLPI) (**Figure 5F**), which were associated with ECM and collagen fibril organization, confirming their matrix CAF (mCAF) phenotype; whereas cells differentiated along state 2, mainly from CAF-COL4A4 and CAF-WNT2, showed upregulated expression of IL-6, IGF1, FBLN1, and the complement genes (C3 and C7) (**Figure 5F**). These markers representing an inflammatory CAF (iCAF) phenotype, which indicates that the CAF-WNT2 and CAF-COL4A4 may engage in immune modulation.

### The phenotypic heterogeneity indicates the multiple sources of CAFs

As the main source of CAFs, NFs can be reprogrammed into CAFs when they are stimulated by tumor cells or other TME components (30, 31). Several receptors involved in cell signal transduction, such as transforming growth factor beta receptor 1/2/3 (TGFBR1/2/3), fibroblast growth factor receptor 1 (FGFR1), and PDGFRβ, were upregulated in CAFs (**Figure 5C**; **Supplementary Figure 5E**).

Epithelial–mesenchymal transition (EMT) processing that is induced by malignant cells is another important mechanism for CAF formation. The EMT-related TFs (EMT-TFs), such as TWIST1 and ZEB1/2, were significantly upregulated both in malignant T cells and CAF subclusters (**Supplementary Figure 5E**). These TFs regulate the expression of EMT markers, such as E-cadherin, and ultimately promote EMT. The IHC experiment results showed that the expression of TWIST1 and MMP2 was significantly increased in tumor stage lesion of MF, indicating an increasing level of EMT and CAF-related matrix remodeling (**Figure 5G**). In addition, the GSVA assays indicated that the identified CAFs showed common activated signatures relating to EMT process, such as mammalian target of rapamycin, FOXO signals, ECM–receptor interaction and regulation of actin cytoskeleton (**Figure 5I**).

These results indicated that CAFs have a high capacity for ECM synthesis and remodeling by producing ECM-degrading proteases, such as matrix metalloproteinases and urokinase-type plasminogen activators. By producing multiple ECM proteins and regulatory molecules, CAFs involved in forming the TME, which promotes tumor initiation, angiogenesis, dissemination, and metastasis.

### CAFs promote tumorigenesis and proliferation through the interaction with malignant T cells

**Figure 6:**
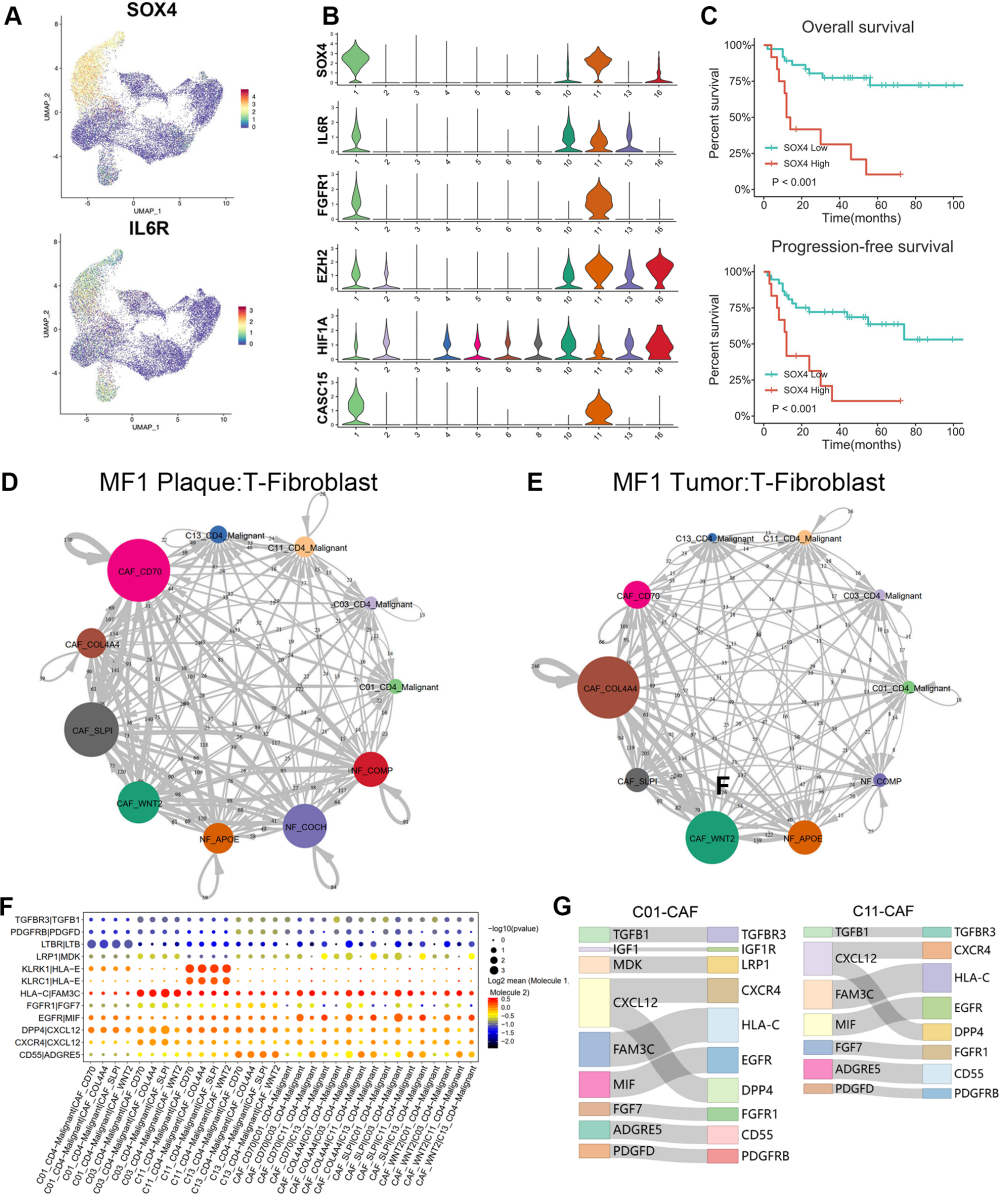
Cell–cell communications between T cells and fibroblasts. **(A)** Expression levels of SOX4 and IL-6R for T-cell subtypes are plotted onto the UMAP plots. **(B)** Violin plots show the expression of specific markers in T-cell subtypes (C01–C06, C08, C10, C11, C13, and C16). **(C)** Overall survival (OS) and progression-free survival (PFS) of patients with MF stratified by SOX4 expression from a publicly available GEO MF cohort of 49 RNA-seq samples via Kaplan–Meier survival analysis. **(D, E)** The interaction number of T cells and fibroblast subpopulations in plaque and tumor stage lesion of MF1 patient. The thickness of the line represents the interaction number between the subpopulations estimated by CellPhoneDB. **(F)** Summary of selected immune-associated ligand–receptor pairs between malignant T cells and CAFs in all samples using CellPhoneDB. The size of each dot denotes the p-value. The color gradient denotes the degree of interaction. **(G)** The major interactions between malignant T cells (C01 and C11) and CAF subtypes.

CAFs were involved in the tumor progression and promoted the aggressiveness of malignant T cells through several pathways. CAFs produced amounts of growth factors, cytokines, and chemokines, including TGFβ, IL-6, and CC-chemokine ligand 2, which may contribute to the immune evasion of malignant cells (**Supplementary Figure 5E**). The elevated expression of IL-6 receptor (IL-6R) and SOX4 were found in the C01, C11 and C13 malignant T subtypes (**Figures 6A, B**). The expression and localization of SOX4, NR4A1, FAP, and IL-6 in corresponding MF skin lesions were confirmed by IHC and IF (**Figures 5G, H**). SOX4 and IL-6 were highly expressed in the tumor stage lesion (**Figure 5G**). The expression of FAP (red), SOX4 (green), and IL-6 (magenta) were also detected by IF imaging (**Figure 5H**). On the other hand, the Hypoxia Inducible Factor 1 Subunit Alpha (HIF-α), Cancer Susceptibility 15 (CASC15) also upregulated in malignant clones (**Figure 6B**).

SOX4 may play a crucial role in the cells transform of NFs to pro-tumorigenic CAFs through the TGF-β/SMAD and Wnt/β-catenin pathway. Additionally, we also suggest that CAFs contribute to the MF progression by pathways of IL-6/Janus kinase 2 (JAK2)/signal transducer and activator of transcription 3 (STAT3)/SOX4/SOX4 and IL-6/HIF-1α/SOX4. Next, we examined the prognostic significance of SOX4 in a publicly available GEO MF cohort. Kaplan–Meier survival analysis showed that higher SOX4 expression was correlated with shorter overall survival (OS) and progression-free survival (PFS) in this GEO MF cohorts (**Figure 6C**), confirming that SOX4 was associated with adverse patient outcomes.

CellphoneDB (21) was performed to investigate the LR interactions between T cells and fibroblasts (**Figures 6D–G**). Results showed the cell–cell communications between malignant T cells and CAFs in samples of different stages (plaque and tumor) from MF1 (**Figures 6D, E**). By further comparing the T-CAF interaction in plaque and tumor stage of MF1, we found that the activated subtype of CAFs varied at different stages of MF. In samples of plaque stage (**Figure 6D**), the CAF-SLPI and CAF-CD70 demonstrate a higher level of activity, whereas, in tumor stage (**Figure 6E**), the interaction was mainly detected between CAF-COL4A4/CAF-WNT2 and malignant T cells.

We further identified the significant LR interactions between C01_CD4–Malignant, C03_CD4–Malignant, C11_CD4–Malignant, C13_CD4–Malignant, and CAFs, such as MDK–LRP1, PDGFD–PDGFRB, CXCL12–CXCR4, TGFBR1–TGFBR3, IGF1–IGF1R, FGF7–FGFR1, CXCL12–DPP4, and MIF–EGFR interactions (**Figure 6F**). CXCL12–CXCR4 and CXCL12–DPP4 were confirmed to involve in promoting MF progression (32, 33). FGF7–FGFR1 was detected in the interaction between CAFs and the main malignant T subtypes (**Figure 6G**). The FGF–FGFR signaling pathway regulates crucial cellular processes and is essential for mesenchymal–epithelial communication (34).

## Discussion

Growing insight into transcriptomic and immunological characteristics of MF provides a more comprehensive understanding of this disease, whereas the origin and evolutionary trajectories of malignant T cells remain controversial and need further elucidation. It was believed that early-stage MF and L-CTCL represented two points on a disease continuum. It was proposed that MF originates from a skin-resident effector memory T-cell subset, whereas Sézary syndrome originates from TCMs (3). Previous studies have found that malignant T cells originated from a monoclonal population derived from mature skin-homing T cells and developed in a multi-step and parallel transformation model (8). Our in-depth analysis of patients with advanced-stage MF revealed the origin and phenotypic changes of malignant T cells during the MF progression. We explored the intercellular interactions between malignant T cells and CAFs, reflecting mechanisms of CAF formation and contribution to disease progression.

To explore the origin of malignant T cells, we tracked the phenotypic changes in T-cell populations during the development and progression of MF. We found the skin TRM cells originate from KLRG1-precursor cells, which are also the origin of the long-lived TCM cell population (27). These precursor cells eventually acquire constitutive surface expression of CD69 and CD103 (ITGAE), which are recognized as key markers of skin TRM cells (27). CD103 and CD69 are essential in tissue residence and differentiation of TRM. As a ligand of E-cadherin, CD103 interact with E-cadherin mediating the attachment between TRM and epithelial cells. CD69-expressing TRM is absent in S1P1 expression, which precludes their ability to detect S1P gradients and exit from peripheral tissues (35). This may explain the epidermotropism of tumor cells in early MF. The CD4+ malignant T cells displayed dual TRM and TCM phenotypes. We suppose these dual phenotype cells including TRM-dominant phenotype and TCM-dominant phenotype cells. During the disease progression, the malignant T cells transformed from TRM-dominant phenotype to the TCM-dominant phenotype. This may explain the reduction or absence of epidermotropism with deeper dermal lymphocytic infiltration in advanced-stage MF.

In addition, we also explored the potential role of the CAFs in promoting the phenotype and metabolic pattern differentiation in tumor cells. CAFs are at the core of cross-communication among various cells in the tumor stroma (36), which release growth factors, cytokines, and chemokines, regulating the biology of tumor cells and other stromal cells via intercellular communication. They also build up and remodel the ECM structure, establishing an invasion-permissive TME (37). NFs can exhibit varying levels of activity in cancer and have the ability to suppress the growth and proliferation of tumor cells (38).

Our data revealed the transcriptional expression patterns of fibroblasts in the TME of MF. Fibroblasts were re-clustered and defined into seven subtypes, of which four subtypes were defined as CAFs with high expression of FAP, S100A4, and PDGFRβ (**Figures 5A, C**). We exhibited the evolution trajectory of seven fibroblast subtypes by the pseudotime analysis (**Figures 5D, E**), which reflects a high inter-tumoral heterogeneity of CAFs in MF and adjacent tissues. The CAFs in each subtype differentiated along divergent trajectory. State 1 mainly consisted of cells from CAF-SLPI and CAF-CD70, whereas state 2 mainly consisted of cells from CAF-WNT2 and CAF-COL4A4 (**Figures 5D, E**). This heterogeneity indicates that these subtypes may belong to distinct CAF populations with different functions.

The formation of CAFs contributes to the development of MF through a variety of mechanisms. CAF-SLPI and CAF-CD70 upregulated in ECM signatures (COL6A3 and POSTN) (**Figure 5F**), which were associated with ECM and collagen fibril organization, confirming their mCAF phenotype. CAF-SLPI and CAF-CD70 CAFs have a high capacity for ECM synthesis and remodeling, which play a critical role in establishing an invasion-permissive TME by producing multiple ECM proteins (such as MMP2) and regulatory molecules. CAF-WNT2 and CAF-COL4A4 upregulated expression of IL-6, IGF1, FBLN1, C3, and C7 (**Figure 5F**), expressing an iCAFs phenotype, indicating that the CAF-WNT2 and CAF-COL4A4 may engage in immune modulation. IL-6 can increase the activity of HIF-1α in tumor cells via STAT3 signaling, and HIF-1α may activate GLUT-1 via the Phosphatidylinositol-3-kinase (PI3K) pathway (39–41), which shifts glucose metabolism from oxidative phosphorylation to anaerobic processes (the Warburg effect) (42–44). This enhances the glucose uptake, causing “metabolic competition” between malignant and reactive T cells, ultimately suppressing the antitumor response (45). Notably, the HIF-1α can also upregulate the expression of SOX4 via an long non-coding RNA (lncRNA) CASC15 (46), which accordance with our results that the malignant subclones showed high levels of HIF-1α, CASC15, and SOX4 (**Figure 6B**). SOX4 regulates the EMT through the activation of EMT-related pathways and multiple EMT TFs (47). Li et al. identified a TGF-β–MTA1–SOX4–EZH2 signaling axis regulating EMT in various cancers and proposed this signaling axis to be a potential therapeutic target in cancer metastasis intervention (48). The SOX4-positive malignant subtypes also upregulated in EZH2 expression (**Figure 6B**), which is consistent with the finding of Li et al. Nasu et al. investigated the immunohistochemical staining SOX4-positive cell score and proposed its diagnostic utility in distinguishing between adult T-cell leukemia/lymphoma and peripheral T-cell lymphoma (49).

IL-6 was reported to skew T-cell differentiation toward Th2 by STAT3 signaling pathway, resulting in the suppression of anti-tumor response (50). Notably, STAT3 has been implicated as a malignant factor in CTCL due to its ability to inhibit apoptosis of tumor cells (51). IL-6 could promote proliferation via JAK2/STAT3/SOX4 pathway in oral squamous cell carcinoma cells (52).

**Figure 7:**
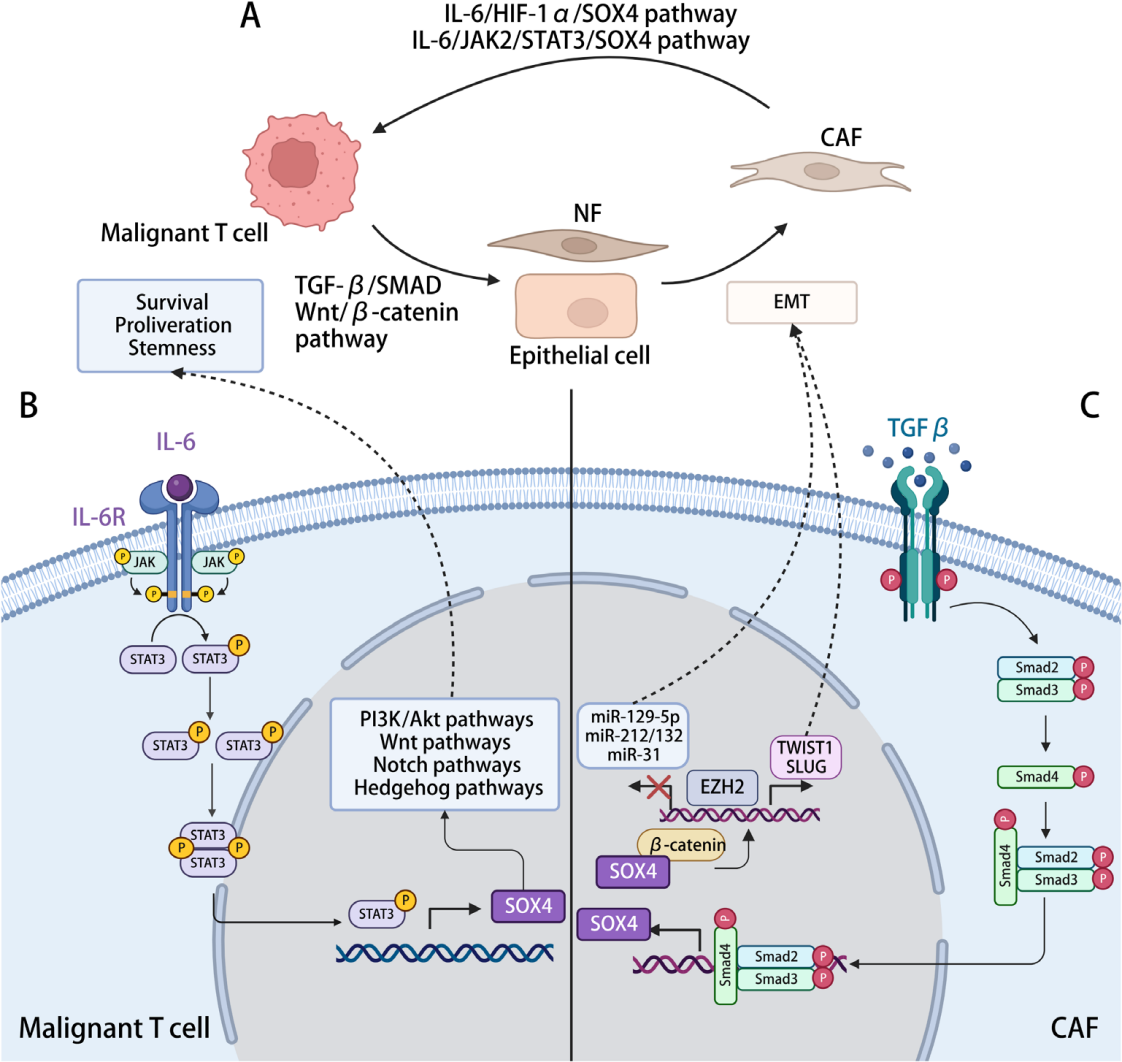
The interaction between malignant T cell and CAFs with SOX4-related mechanisms in the process. **(A)** Malignant T cells in MF induce the activation and formation of CAFs, which, in turn, modulating cancer progression. **(B)** Activation of SOX4 expression in malignant T cells. IL-6 generated by CAFs, activating the JAK2/STAT3 pathway in malignant T cells, positively regulated the transcription of SOX4, further activating the PI3K/Akt, Wnt/β-catenin, Notch, and Hedgehog signaling pathway, hence promoting tumorigenesis. **(C)** Activation of SOX4 expression in CAFs. TGF-β generated by malignant cells activates the TGF-β signaling in NFs or epithelial cells, leading to the phosphorylation of SMAD2/3, which promotes SOX4 expression after translocating to the nucleus. By interacting with β-catenin and ERG, SOX4 induces the expression of EZH2, which might directly regulate TWIST1 and modulating the EMT procession, reprograming other cells into CAFs. The SOX4–EZH2 axis also modifies the promoter region of tumor-suppressor microRNAs (miRNAs), including miR-31, miR-212/132, and miR-129-2. By repressing the transcription of these miRNAs, SOX4 involved in the inhibition of cell proliferation and migration.

There may be mutual positive feedback of the crosstalk between tumor cells and CAFs during the MF development. The malignant T cells promote the CAF formation through the SOX4-associated EMT, such as TGF-β–MTA1–SOX4–EZH2 pathway. The CAFs, in turn, improve the invasiveness and metastasis of the malignant T cells through the IL-6/JAK2/STAT3/SOX4 or IL-6/HIF-1α/SOX4 pathway. Conclusively, SOX4 may be a critical regulatory gene of this positive feedback loop, and targeting SOX4 may disrupt the interactions between neoplastic clones and TME, reversing the immunosuppressive TME (**Figure 7**).

Cellphone DB showed significant LR interactions between fibroblasts and T cells, such as FGF7–FGFR1, MDK–LRP1, and PDGFD–PDGFRB interactions (**Figure 6G**). The malignant T-cell subtypes showed a strong intercellular interaction with CAFs in the MDK–LRP1 axis (**Figure 6G**). We hypothesized that MDK secreted by malignant T cells could interact with LRP1 on the surface of CAFs to upregulate the proliferation of CAFs. The enriched MDK–LRP1 signal may serve as a sign of CAF activation to stimulate downstream pathways for promoting tumor invasion and, thus, may be a potential early biomarker of MF progression.

FGF7–FGFR1 was detected in the interaction between CAFs and most malignant T subtypes (**Figure 6G**). The FGF–FGFR signaling pathway regulates crucial cellular processes and is essential for mesenchymal–epithelial communication (34). CAFs activate the FGF signaling and promote the proliferation, migration, and invasion of cancer cells (53). Combination of FGF signaling and TGF-β promotes CAF formation with migratory and proliferative properties. It was also reported that CAF function was regulated by FGF and TGF-β signaling (54). Further studies are necessary to confirm the SOX4-associated pathogenesis and the intercellular interactions between malignant T cells and CAFs in MF.

The study limitation of included the cohort of only two patients with MF and matched HC. However, for each patient with MF, we collected samples with different stages as well as the corresponding adjacent non-lesional skin, which is also meaningful in revealing the evolutionary trajectory of tumor cells. In addition to survival analyses in the GEO cohorts and IHC experiment, more validation experiments are needed to further verify the findings.

In summary, this study revealed that the malignant T cells in MF may originate from TRM precursor cells, with subclones displaying dual TRM and TCM cell phenotypes. During the disease progression, the malignant T cells transformed from TRM-dominant phenotype to the TCM-dominant phenotype, which associated with more aggressive biological behavior. Our study also provided new insights into the intercellular interactions between malignant T cells and CAFs. The activated LR pairs, such as FGF7–FGFR1 and MDK–LRP1, involved in the proliferation of CAFs and promotion of tumor invasion. We further illustrated the SOX4-associated pathogenesis in MF. SOX4 serves as critical regulatory genes of the positive feedback loops of the interaction between malignant T cells and CAFs. This study provides a comprehensive understanding on the molecular mechanism of MF and advances the knowledge on novel targets for MF treatment.

## Data Availability

The data presented in the study are deposited in the Genome Sequence Archive (Genomics, Proteomics & Bioinformatics 2021) in National Genomics Data Center (Nucleic Acids Res 2022), China National Center for Bioinformation/Beijing Institute of Genomics, Chinese Academy of Sciences (GSA-Human) repository, accession number HRA007111.
