# Supplementary material for: The cancer-associated fibroblasts interact with malignant T cells in mycosis fungoides and promote the disease progression
Source: Front Immunol. 2025 Feb 3;15:1474564. doi: 10.3389/fimmu.2024.1474564 (PMC11830738; doi:10.3389/fimmu.2024.1474564)
Supplement: Supplementary file 1 [file DataSheet1.docx]

**Supplementary figures and figure legends**

The cancer-associated fibroblasts interact with malignant T cells in mycosis fungoides and promote the disease progression

**
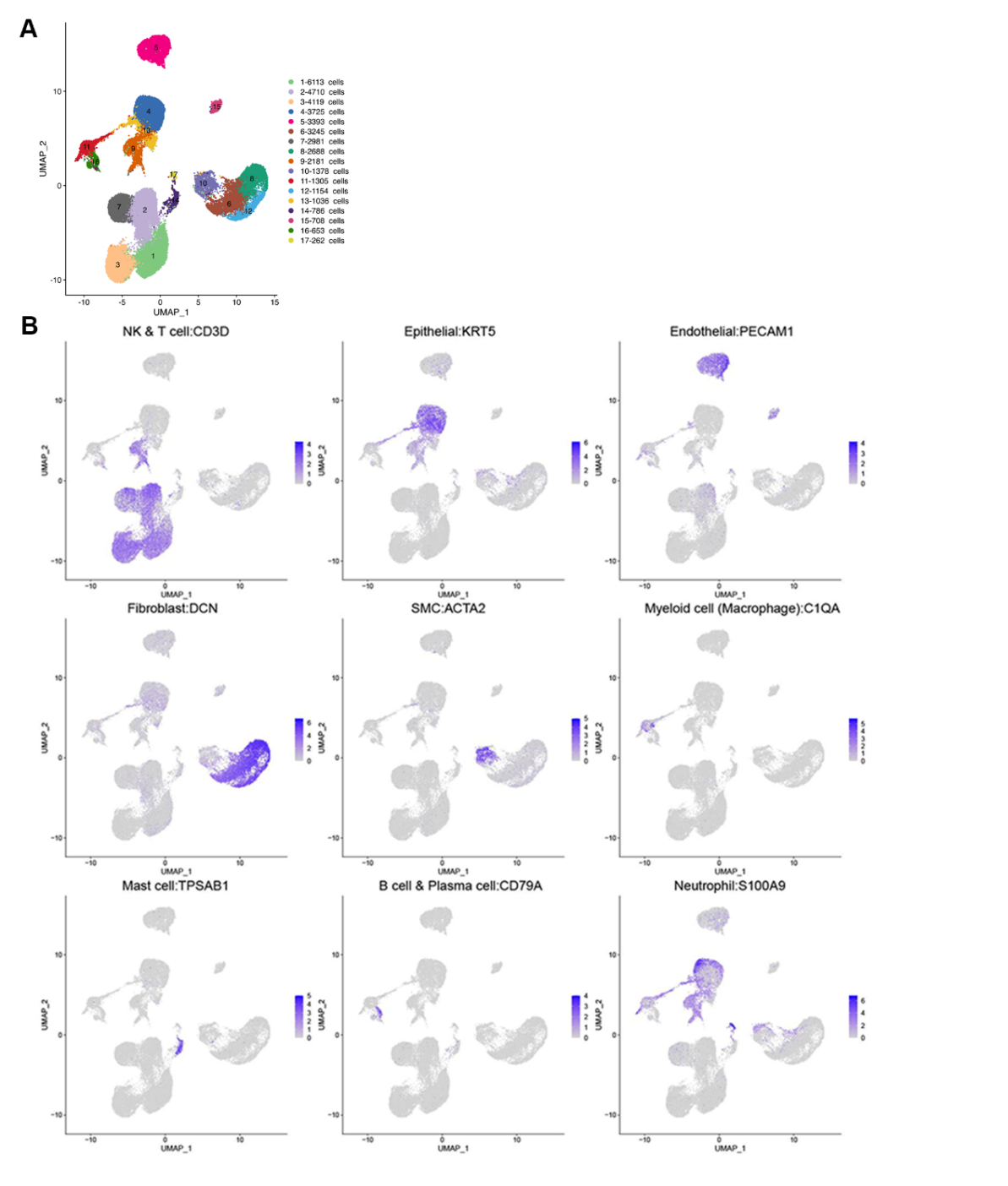
**

**Supplementary Figure. 1 Distribution patten of cell clusters from CTCL patients and HC visualized by UMAP plots.**

**A** Clustering of cells from CTCL patients and HC.

**B** UMAP plots showing the expression of major discriminative marker genes for cell types identification of cells from CTCL patients and HC. The color scale represents normalized expression. Gray to blue: low to high expression.


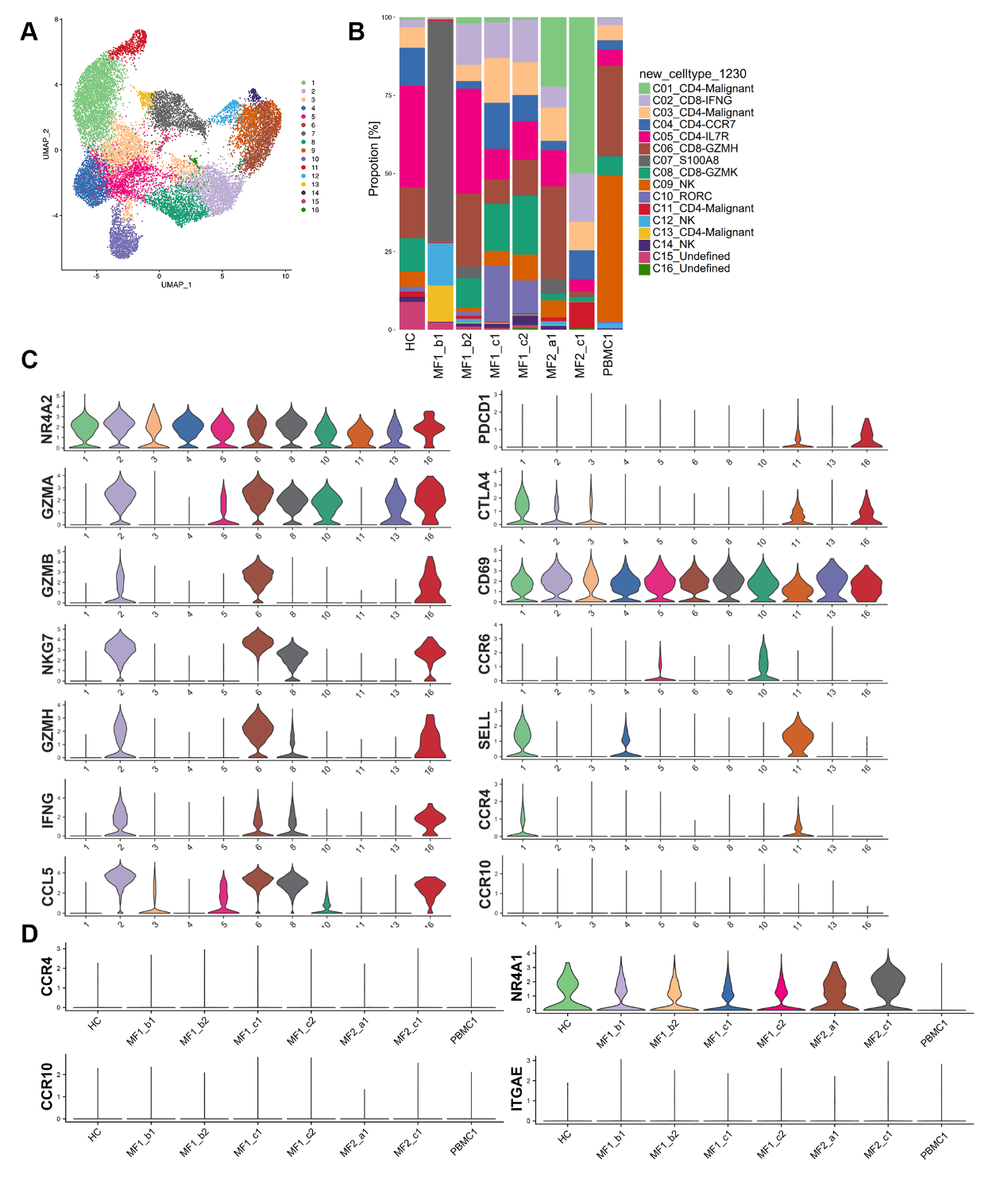
**Supplementary Figure. 2 Sub-clustering of T cells from the CTCL patients and HC.**

**A** T cells were re-clustered into 16 subclusters.

**B** Stacked histogram showing the percentage of cells from various T subtypes in each sample.

**C** Violin plots show the expression of specific markers in T cell subtypes (C01-C06, C08, C10, C11, C13, C16).

**D** Violin plots show the expression of skin homing molecules genes (CCR4 and CCR10) and tissue-resident associated genes (NR4A1, CD103) in T cells subtypes of each sample.


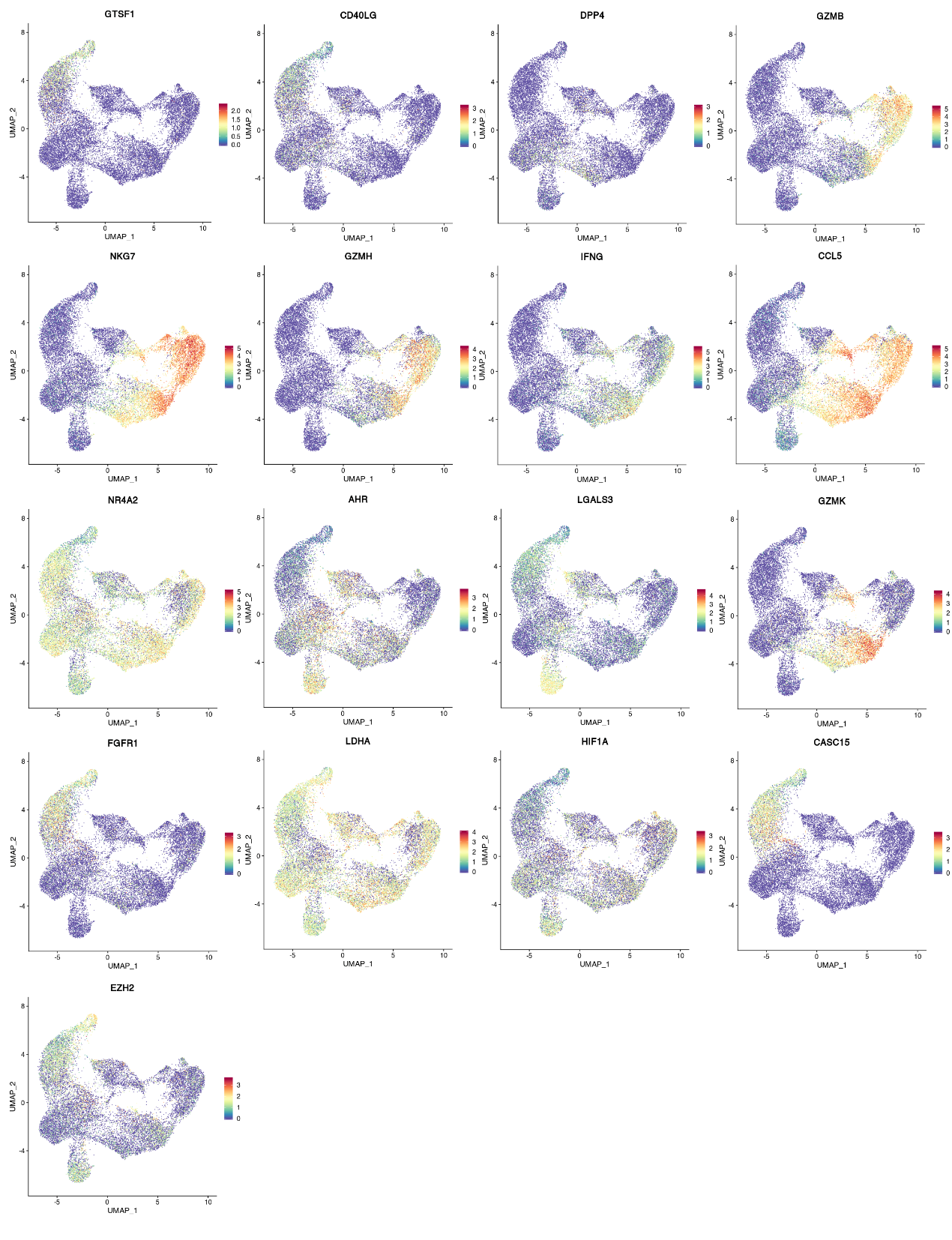


**Supplementary Figure. 3** UMAP plots showing the expression of selected marker genes in T cell subtypes.


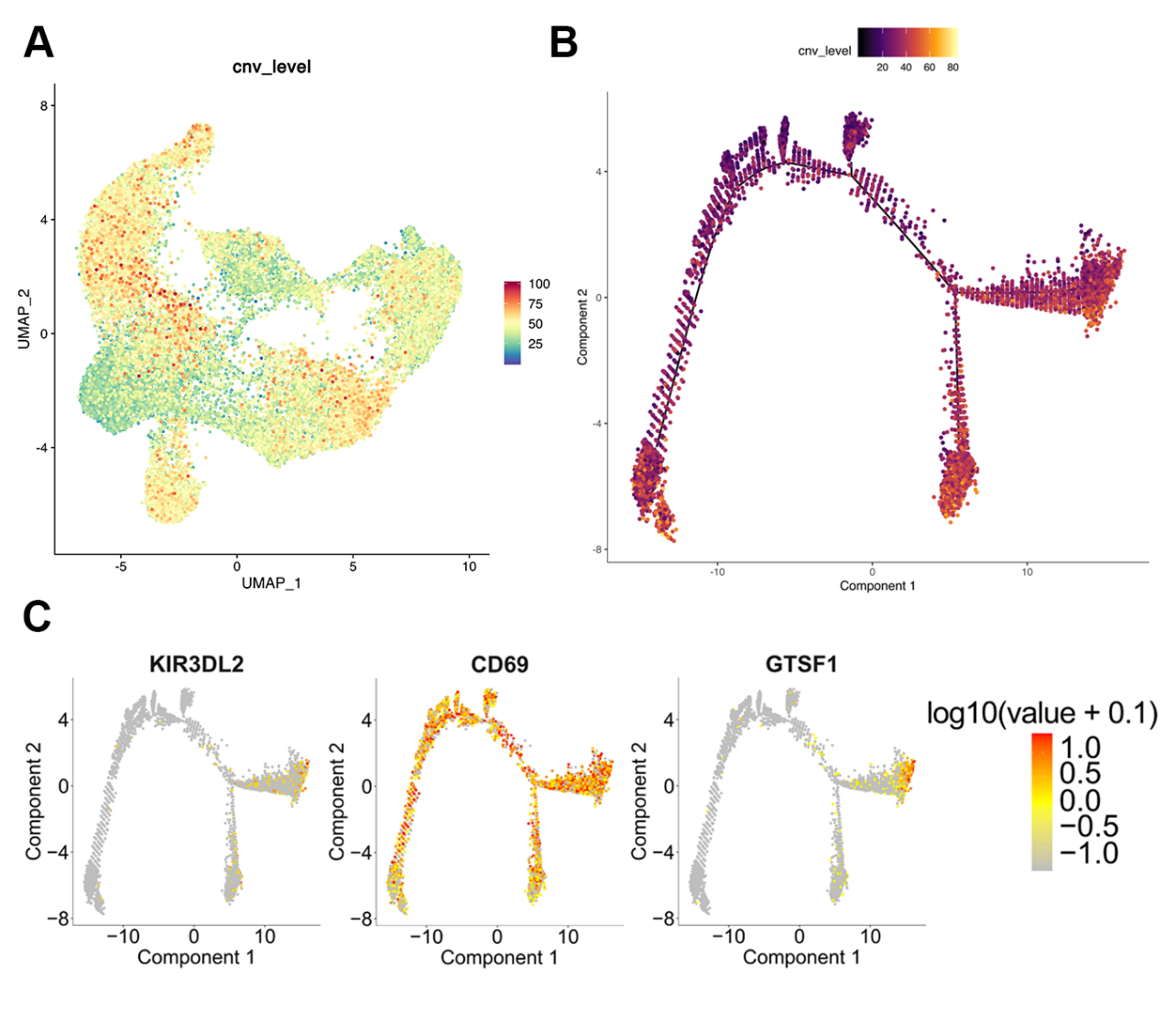


**Supplementary Figure. 4** CNVs levels overlaid on the UMAP plots and pseudotime trajectory of T cells.

**A** CNVs levels of T subtypes were plotted onto the UMAP plots.

**B** CNVs levels of CD4+ T cells overlaid on the pseudotime trajectory.

**C** Trajectory plots with the expression of respective malignant T cell differentiation-associated genes, highest expression in red, lowest expression in grey.


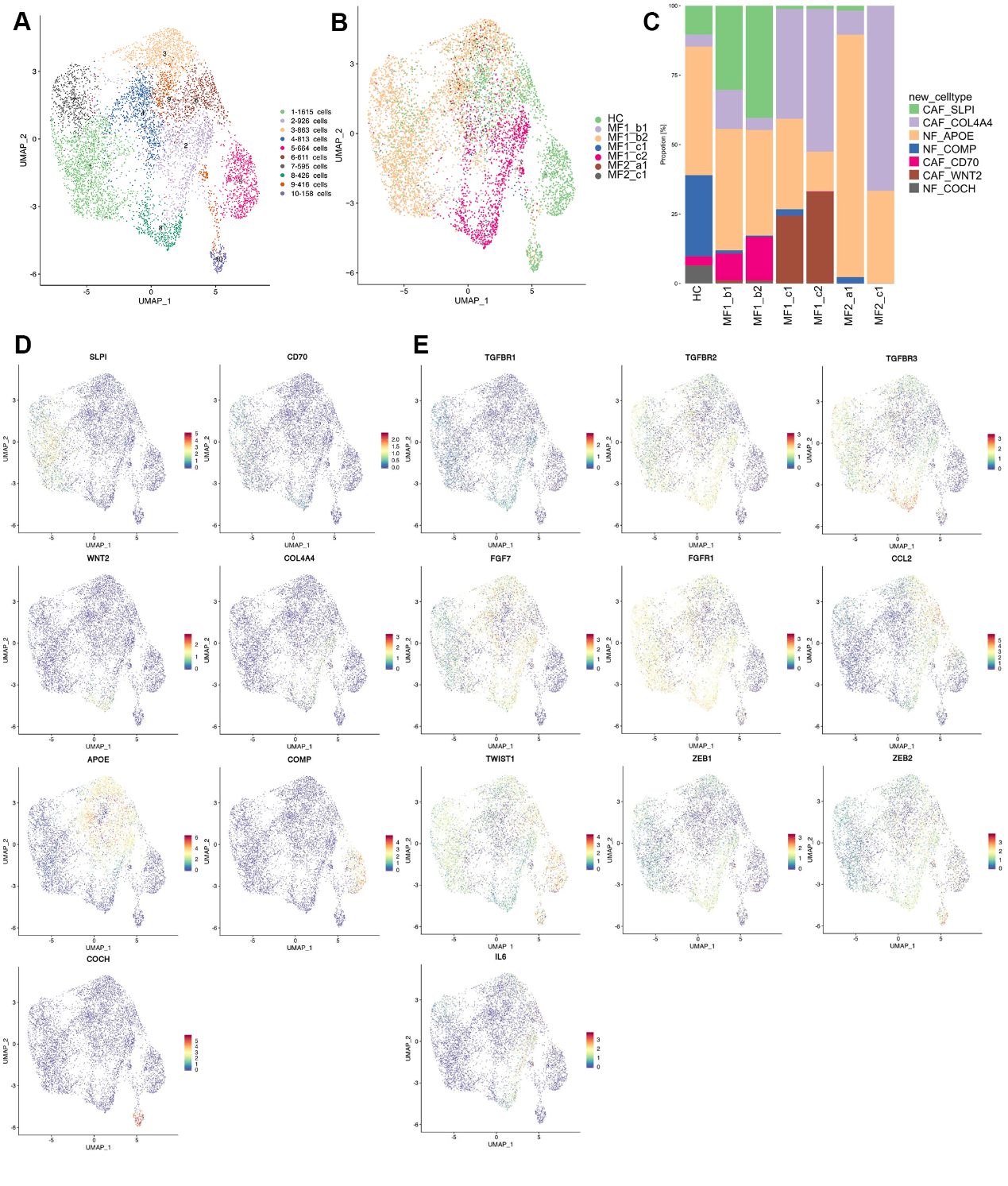


**Supplementary Figure. 5** Transcriptional patterns of fibroblasts from CTCL patients and HC.

**A** UMAP visualization of fibroblasts from skin samples of two CTCL patients and one HC.

**B** UMAP plot for fibroblasts split by 7 skin samples from two CTCL patients and one HC.

**C** Stacked histogram showing the percentage of cells from various cell types in each sample.

**D** UMAP plots showing the expression of major discriminative marker genes for cell types identification of fibroblast subtypes from CTCL patients and HC.

**E** UMAP plots showing the expression of selected marker genes in fibroblast subtypes.
